# Pigs as Natural Hosts of *Dientamoeba fragilis* Genotypes Found in Humans

**DOI:** 10.3201/eid1805.111093

**Published:** 2012-05

**Authors:** Simone M. Cacciò, Anna Rosa Sannella, Elisabetta Manuali, Fabio Tosini, Marco Sensi, Daniele Crotti, Edoardo Pozio

**Affiliations:** Istituto Superiore di Sanità, Rome, Italy (S.M. Cacciò, A.R. Sannella, F. Tosini, E. Pozio);; Istituto Zooprofilattico Sperimentale dell’Umbria e delle Marche, Perugia, Italy (E. Manuali, M. Sensi, D. Crotti)

**Keywords:** Parasites, Dientamoeba fragilis, pigs, zoonoses, protozoa, humans, genotypes

## Abstract

*Dientamoeba fragilis* is a common intestinal parasite in humans. Transmission routes and natural host range are unknown. To determine whether pigs are hosts, we analyzed 152 fecal samples by microscopy and molecular methods. We confirmed that pigs are a natural host and harbor genotypes found in humans, suggesting zoonotic potential.

## Abstract 2

The world is home to more than 1 billion pigs, which produce large quantities of feces. We know that some organisms in pig feces can cause human disease, and now we might have another to add to the list. Little is known about where the common intestinal parasite *Dientamoeba fragilis* comes from and how it is spread. However, recent molecular analysis confirmed that the organism found in pigs is indeed the same as the one found in humans. Therefore, pigs (or their feces) might be a source of this parasitic infection in humans.

The flagellated protozoan *Dientamoeba fragilis* is one of the most common parasites in the intestinal tract of humans (*1*). Infection is highly prevalent in economically developing regions and in industrialized countries (*1**,**2*). Infected persons often show no symptoms, but a pathogenic role for this parasite has been reported recently in humans and gorillas (*2**–**4*). Little is known about transmission routes of this parasite, and a transmissible stage (e.g., a cyst) has not been described (*1**,**5*). Molecular characterization of human isolates based on sequence analysis of ribosomal genes revealed 2 genotypes (1 and 2), with genotype 1 predominating worldwide (*6**,**7*).

Other than humans, few animal hosts of *D. fragilis* have been reported. Surveys of mammals and birds have identified only nonhuman primates (gorillas, macaques, and baboons) as natural hosts (*8**,**9*). Recently, however, a high prevalence of infection (43.8%) has been reported in pigs in Italy (*10*). To determine whether pigs are a host of *D. fragilis*, we analyzed fecal samples from 152 pigs in Italy by microscopy and molecular methods.

## The Study

During June–August 2010, a total of 152 fecal samples were collected from the rectums of piglets (age 1–3 months; weight 6–24 kg), fattening pigs (age 3–4 months; weight 25–50 kg), and sows (age 1–2 years; weight 180–250 kg). The pigs were raised in 6 farrow-to-finish farms, 2 fattening farms, and 1 weaner indoor farm of central Italy (7 farms in the Umbria region and 2 farms in the Marche region). Pig fecal samples from 7 of the 9 farms were available for molecular analysis. Fecal samples from 21 pig farmers were collected from 5 of the 9 farms, 17 of which were available for molecular analysis.

**Figure 1:**
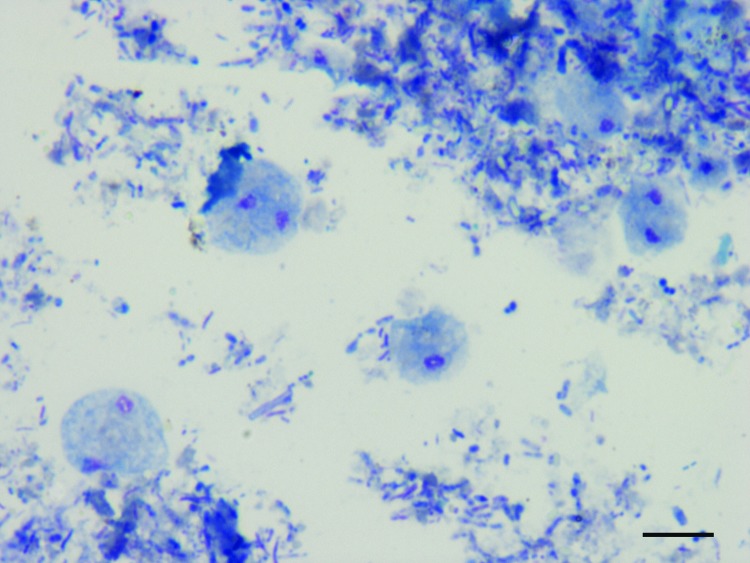
*Dientamoeba fragilis* trophozoites in a smear of pig feces after Giemsa staining, Italy, 2010–2011. Scale bar = 10 μm.

Microscopic diagnosis of *D. fragilis* was based on visualization of pleomorphic trophozoites, ranging in size from 4 µm to 20 µm, with fragmented chromatin and pale gray-blue finely vacuolated cytoplasm after Giemsa staining (Figure 1). DNA was extracted directly from 200 mg of feces by using the QIAamp DNA stool minikit (QIAGEN, Hilden, Germany). Reference *D. fragilis* DNA of genotype 1 (strains 379 and 1085) was used as a positive control.

A TaqMan real-time PCR that targets the 5.8 S ribosomal locus was performed in a LightCycler 480 apparatus (Roche Diagnostics GmbH, Mannheim, Germany) as described (*11*). For the 18S rRNA gene, a published assay (*12*) was used to amplify a 662-bp fragment, followed by amplification of a 366-bp fragment with newly designed primers DF322For (5′-GAGAAGGCGCCTGAGAGATA-3′) and DF687Rev (5′-TTCATACTGCGCTAAATCATT-3′). For the internal transcribed spacer 1 (ITS1) region, a nested PCR protocol was developed. In the primary reaction, the forward primer ssu2 (*13*) and the reverse primer Df-ITSRev (5′-GCGGGTCTTCCTATATAAACAAGAACC-3′) were used, whereas the forward primer Df-ITSnesFor (5′-ATACGTCCCTGCCCTTTGTA-3′) and the reverse primer Df-ITSnesRev (5′-GCAATGTGCATTCAAAGATCGAAC-3′) were used in the nested PCR. PCR products were purified and sequenced on both strands. The sequences were assembled by using SeqMan II (DNASTAR, Madison, WI, USA) and compared with those available in public databases by using BLAST (http://blast.ncbi.nlm.nih.gov/Blast.cgi). Sequences from this study were submitted to GenBank under accession nos. JQ677147–JQ677168.

The microscopic examination showed that 52 of the 74 piglets, 11 of the 14 fattening pigs, and 8 of the 64 sows were positive for *D. fragilis* (Table 1). More trophozoites were observed in fecal samples from piglets, suggesting a higher susceptibility of young animals to infection (data not shown). The microscopic analysis also showed *Blastocystis* spp. (in 42% of pigs), *Endolimax nana* protozoa (32%), *Iodoamoeba buetschli* protozoa (25%), and other flagellates (4.5%). Of the 21 samples from pig farmers, 4 from farmers working on 2 farms were positive for *D. fragilis* by microscopy (Table 1).

**Table 1:** Prevalence of *Dientamoeba* *fragilis* protozoa in pig and human fecal samples after microscopy and Giemsa staining, Italy, 2010–2011

Farm	Herd type	No. samples positive/no. tested*			
		Piglet	Fattening pig	Sow	Human
1	Weaner production	10/10	NA	1/10	NA
2	Farrow-to-finish	9/10	NA	3/10	0/4
3	Farrow-to-finish	10/10	7/10	0/10	2/8
4	Farrow-to-finish	1/10	NA	0/10	NA
5	Farrow-to-finish	4/10	NA	0/10	0/2
6	Farrow-to-finish	4/10	NA	1/10	NA
7	Fattening	NA	NA	3/4	NA
8	Fattening	10/10	NA	NA	2/3
9	Farrow-to-finish	4/4	4/4	NA	0/4
	Total	52/74	11/14	8/64	4/21

*NA, sample not available.

Molecular techniques were applied to 38 pig fecal samples, specifically 24 samples positive by microscopy from 6 farms and 14 samples negative by microscopy from 2 farms, and to all 17 human fecal samples. A comparison of human and pig samples collected from the same farm was possible for farms 2, 3, and 5 (Table 2). Using real-time PCR, all 24 positive pig samples were amplified, with cycle threshold values ranging from 30 to 34, whereas none of the 14 negative samples were positive to this assay (Table 2). However, because no inhibition controls were run, false-negative results cannot be ruled out. Of the 17 human fecal samples, 13 were positive with cycle threshold values of 29–40. The sequence analysis of 15 amplified products (11 from pigs and 4 from humans) showed 100% homology with *D. fragilis* genotype 1 (Table 2). Genotype 2 was not found in any of the samples from pigs or humans.

**Table 2:** Results of the molecular tests for *Dientamoeba* *fragilis* applied to pig and human fecal samples, Italy, 2010–2011*

Sample	Farm	Microscopy	5.8S			18S			ITS1	
			Real-time PCR	Sequence		PCR	Sequence		PCR	Sequence
P21	2	+	+	ND		–	ND		–	ND
P26	2	+	+	*D. fragilis*		+	*D. fragilis†*		+	*D. fragilis‡*
P27	2	+	+	*D. fragilis*		–	ND		+	unclassified
P37	2	+	+	ND		–	ND		–	ND
P42	3	+	+	*D. fragilis*		+	*D. fragilis†*		+	*D. fragilis*§
P44	3	+	+	*D. fragilis*		–	ND		+	flagellate
P45	3	+	+	ND		–	ND		–	ND
P50	3	+	+	ND		–	ND		–	ND
P52	3	+	+	*D. fragilis*		–	ND		+	unclassified
P54	3	+	+	*D. fragilis*		–	ND		+	flagellate
P56	3	+	+	*D. fragilis*		+	*D. fragilis†*		+	unclassified
P59	3	+	+	*D. fragilis*		+	*D. fragilis†*		+	unclassified
P60	3	+	+	ND		–	ND		–	ND
P71	4	+	+	ND		–	ND		–	ND
P75	4	+	+	ND		–	ND		–	ND
P91	5	+	+	ND		–	ND		–	ND
P93	5	+	+	*D. fragilis*		+	*D. fragilis†*		+	unclassified
P 97	5	+	+	*D. fragilis*		+	*D. fragilis†*		+	flagellate
P111	6	+	+	ND		–	ND		–	ND
P113	6	+	+	ND		–	ND		–	ND
P116	6	+	+	ND		–	ND		–	ND
P122	6	+	+	*D. fragilis*		–	ND		+	flagellate
P131	6	+	+	ND		–	ND		–	ND
P133	7	+	+	ND		–	ND		–	ND
Pig 1	1	–	–	ND		+	*Trichomitus*¶		–	ND
Pig 2	1	–	–	ND		–	ND		–	ND
Pig 3	1	–	–	ND		+	*Trichomitus*¶		–	ND
Pig 4	1	–	–	ND		–	ND		–	ND
Pig 5	1	–	–	ND		+	*Trichomitus*¶		–	ND
Pig 6	1	–	–	ND		–	ND		–	ND
Pig 7	1	–	–	ND		–	ND		–	ND
Pig 8	1	–	–	ND		–	ND		–	ND
Pig 9	1	–	–	ND		–	ND		–	ND
Pig 10	1	–	–	ND		–	ND		–	ND
DF-P1	6	–	–	ND		–	ND		–	ND
DF-P2	6	–	–	ND		–	ND		–	ND
DF-P3	6	–	–	ND		–	ND		–	ND
DF-P4	6	–	–	ND		–	ND		–	ND
H1	2	–	+	*D. fragilis*		+	*D. fragilis†*		–	ND
H2	2	–	–	ND		–	ND		–	ND
H3	2	–	+	ND		–	ND		–	ND
H4	2	–	+	ND		+	*D. fragilis†*		–	ND
H5	5	–	+	ND		–	ND		–	ND
H6	5	–	+	ND		+	*D. fragilis†*		–	ND
H7	3	–	+	ND		+	*D. fragilis†*		–	ND
H8	3	–	+	ND		–	ND		–	ND
H9	3	–	–	ND		–	ND		–	ND
H10	3	+	+	ND		+	*D. fragilis†*		–	ND
H11	3	+	+	*D. fragilis*		+	*D. fragilis†*		–	ND
H12	3	–	+	ND		–	ND		–	ND
H13	3	–	+	ND		–	ND		–	ND
H14	3	–	–	ND		–	ND		–	ND
H15	8	+	+	*D. fragilis*		+	*D. fragilis†*		+	*D. fragilis#*
H16	8	+	–	ND		–	ND		–	ND
H17	9	–	+	*D. fragilis*		+	*D. fragilis†*		+	*D. fragilis#*

*ITS, internal transcribed spacer; ND, not done. †100% identity to AY730405. ‡100% identity to DQ223443, DQ223447, and DQ223455. §100% identity to DQ223448 and DQ223453. ¶96% identity to AF124610. #100% identity to DQ223442, DQ223450, DQ223452, DQ223454, DQ223456, and DQ167586.

**Figure 2:**
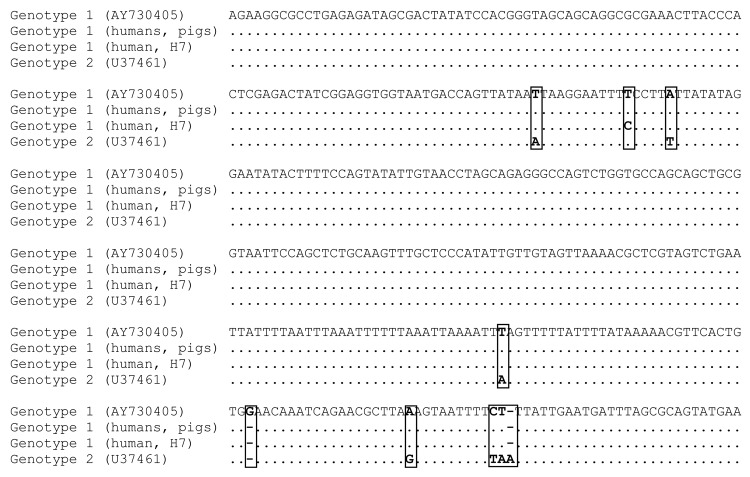
Multiple alignment of the 366-bp fragment of the 18S rRNA gene from *Dientamoeba fragilis* genotypes 1 and 2. Dot indicates identical nucleotides. Dashes indicate insertion or deletion. Nucleotide differences are presented in boxes.

Next, a 366-bp fragment of the 18S rRNA gene was analyzed. In this fragment, genotypes 1 and 2 can be distinguished by 8 substitutions or insertions or deletions (Figure 2), which were further confirmed by sequencing the entire 18S rRNA gene from 2 reference isolates and 2 human isolates from this study. Amplification was obtained from 6 of the 24 positive pig samples and from 8 of the 17 human samples. Genotype 1 was identified in all samples (Figure 2). One human isolate (H7) showed a single nucleotide substitution in the fragment sequenced (Figure 2). Sequences from 3 microscopically negative pig samples (all from farm 1) had a high homology (96%) with *Trichomitus batrachorum*, a flagellate of reptiles, although the sequence could originate from *T. rotunda*, a flagellate of pigs that has not been described at the molecular level.

Last, we studied the more variable ITS1 locus. Amplification was obtained from 11 of the 24 pig samples (Table 2), but only 2 sequences could be clearly identified as *D. fragilis*. Four sequences showed homology (80%) with flagellates from different vertebrate classes whereas the remaining 5 sequences were excluded because of insufficient quality. The 2 *D. fragilis* sequences from pigs showed 100% homology with sequences from human isolates from the United Kingdom (Table 2), further supporting the presence of genotype 1 in these 2 hosts. A direct comparison of ITS1 sequences from humans and pigs from a single farm in Italy was not possible because *D. fragilis* was amplified from only 2 human samples from 2 farms from which no pig samples were available. The analysis of ITS1 from the 2 human isolates showed full identity to human isolates from the Netherlands and the United Kingdom (Table 2).

## Conclusions

Considering the size of the world’s pig population (>1 billion), the close contact between pigs and humans in many parts of the world, and the difficulties in the proper management of pig fecal waste, the role of these animals as reservoirs of zoonotic pathogens must be carefully evaluated. We demonstrated that pigs are hosts of *D. fragilis*, on the basis of molecular analysis of 3 fragments in the ribosomal cluster. Sequence analyses of fragments of the 18S and 5.8S rRNA genes showed genotype 1 in isolates collected in the same farm from humans and pigs, suggesting the potential for zoonotic transmission of this parasite. If a transmissible cyst stage exists, then environmental contamination with pig feces should be considered a key factor in the transmission of this parasite. Pigs also are a fascinating animal model to elucidate the life cycle of this elusive parasite.
